# Cell Shape and Durotaxis Explained from Cell-Extracellular Matrix Forces and Focal Adhesion Dynamics

**DOI:** 10.1016/j.isci.2020.101488

**Published:** 2020-08-22

**Authors:** Elisabeth G. Rens, Roeland M.H. Merks

**Affiliations:** 1 Scientific Computing, CWI, Science Park 123, 1098 XG Amsterdam, the Netherlands; 2 Mathematics Department, University of British Columbia, Mathematics Road 1984, Vancouver, BC V6T 1Z2, Canada; 3 Mathematical Institute, Leiden University, Niels Bohrweg 1, 2333 CA Leiden, the Netherlands

**Keywords:** Biological Sciences, Cell Biology, Biophysics, In Silico Biology, Biomaterials, Structural Biology, Biochemistry, Biocomputational Method

## Abstract

Many cells are small and rounded on soft extracellular matrices (ECM), elongated on stiffer ECMs, and flattened on hard ECMs. Cells also migrate up stiffness gradients (durotaxis). Using a hybrid cellular Potts and finite-element model extended with ODE-based models of focal adhesion (FA) turnover, we show that the full range of cell shape and durotaxis can be explained in unison from dynamics of FAs, in contrast to previous mathematical models. In our 2D cell-shape model, FAs grow due to cell traction forces. Forces develop faster on stiff ECMs, causing FAs to stabilize and, consequently, cells to spread on stiff ECMs. If ECM stress further stabilizes FAs, cells elongate on substrates of intermediate stiffness. We show that durotaxis follows from the same set of assumptions. Our model contributes to the understanding of the basic responses of cells to ECM stiffness, paving the way for future modeling of more complex cell-ECM interactions.

## Highlights

• A cellular Potts model is extended with the mechanical response of focal adhesions
• Mechanical cell-ECM reciprocity leads to cell spreading, elongation, and durotaxis
• Cell-generated planar stresses may cooperatively strengthen focal adhesion patches
• Predicted durotaxis speed increases with gradient steepness until saturation point

## Graphical Abstract

**Figure:**
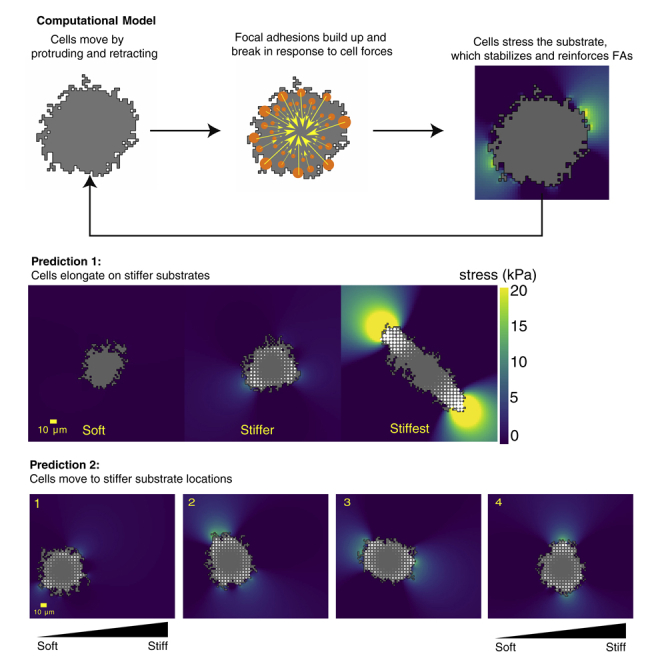

## Introduction

Mechanical interactions between cells and the extracellular matrix (ECM) are crucial for the formation and function of tissues. By sensing and responding to physical forces in the ECM, cells change their shape and migrate to other locations. The shape of a wide range of mammalian cell types depends on the stiffness of the ECM. *In vitro*, cells cultured on soft, two-dimensional ECM substrates become relatively small and round, whereas on stiffer ECMs the cells assume elongated shapes. On highly rigid substrates such as glass, cells spread out and flatten. This behavior has been observed for a wide range of cell types, including endothelial cells (Califano and Reinhart-King, 2010), fibroblasts (Ghibaudo et al., 2008; Pelham and Wang, 1997; Prager-Khoutorsky et al., 2011), smooth muscle cells (Engler et al., 2004), and osteogenic cells (Mullen et al., 2015). Cells tend to migrate toward stiffer parts of the ECM, a phenomenon known as durotaxis. Such behavior also occurs for a wide range of mammalian cell types, including fibroblasts (Lo et al., 2000), vascular smooth muscle cells (Isenberg et al., 2009), mesenchymal stem cells (Vincent et al., 2013), and neurons and glioma cells (Bangasser et al., 2017). Here, we show that a single model suffices to explain (a) increased cell area on stiffer substrates, (b) cell elongation on substrates of intermediate stiffness, and (c) durotaxis.

It is still not completely understood what molecular mechanisms regulate such cellular response to ECM stiffness (Jansen et al., 2015). Cells are able to sense matrix stiffness through focal adhesions (FAs). FAs are multimolecular complexes consisting of integrin molecules that mediate cell-ECM binding and force transmission and an estimated further 100 to 200 protein species that strongly or more loosely associate with FAs (Bershadsky et al., 2003a; Winograd-Katz et al., 2014; Zaidel-Bar et al., 2007). Among these are vinculin and talin, which bind integrin to actin stress fibers.

FAs dynamically assemble and disassemble, at a rate that is regulated biochemically and mechanically. The disassembly rate is highest on soft ECMs and is lower on stiffer ECMs (Pelham and Wang, 1997) such that FAs stabilize more easily on stiffer ECMs. This mechanosensitivity of FA dynamics is regulated by talin and p130Cas, proteins that change conformation in response to mechanical force (Bershadsky et al., 2003a; Jansen et al., 2015). For instance, stretching the structural protein talin reveals vinculin-binding sites, allowing additional vinculin to bind to FAs (Rio et al., 2009) and stabilize the FA (Gallant et al., 2005). Furthermore, integrins such as α5-β1, behave as so-called “catch-bonds” (Kong et al., 2009), whose lifetime increases under force (Dembo et al., 1988).

Interestingly, manipulations of FA formation affect both cell spreading and cell motility. Systematic knock-down of protein-tyrosine kinases involved in FA formation and traction force development reduces the ability of fibroblasts to elongate in a substrate-stiffness-dependent manner (Prager-Khoutorsky et al., 2011). FA assembly and disassembly has also been associated with cell migration and cell orientation (Broussard et al., 2008; Chen et al., 2013; Kim et al., 2013; Plotnikov et al., 2012) in response to the ECM. Hence, the mechanosensitive growth of FAs is key to our understanding of cellular responses to ECM stiffness, but how FAs and cytoskeletal force generation work together to regulate cell spreading, cell shape, and durotaxis is still to be elucidated.

Previous mathematical models for mechanosensitivity have proposed explanations for cell spreading, cell elongation, or durotaxis, but did not explain the three phenomena in unison. A central idea, dynamic mechanoreciprocity (Bissell et al., 1982; van Helvert et al., 2018), posits that properties of the ECM affect the mechanical and/or biochemical properties of the cell, that, in turn, change the local environment. For example, Ni et al. (Ni and Chiang, 2007) assumed that cells pull more strongly due to mechanical resistance of the matrix (Ni and Chiang, 2007), resulting in spreading of the cell; Shenoy et al. (Shenoy et al., 2015) proposed that stresses in the ECM can polarize the cytoskeleton. Cell spreading is also the main process addressed in several kinetic models of FAs (Deshpande et al., 2008; Ronan et al., 2014; Stolarska and Rammohan, 2017; Vernerey and Farsad, 2014). In these models it is assumed that the greater stress that FAs experience on stiffer substrates accelerate the formation of stress fibers and enable yet greater cell forces to be applied. The resulting positive feedback loop drives cells to spread out on stiffer substrates. More recently, such frameworks have been expanded with thermodynamic considerations (McEvoy et al., 2018; Shishvan et al., 2018). The prevalent idea of durotaxis is that increased stabilization of FAs at the stiff side of the cell leads to increased traction force (Dokukina and Gracheva, 2010), modified stress fiber dynamics (Harland et al., 2011; Lazopoulos KA, 2008), cell speed, and/or polarization (Kim et al., 2018; Malik and Gerlee, 2019; Novikova et al., 2017; Stefanoni et al., 2011; Yu et al., 2017), or other cell dynamics (Allena et al., 2016; Aubry et al., 2015; Shenoy et al., 2015).

Another explanation for cell anisotropy and durotaxis is based on the motor-clutch hypothesis (Chan and Odde, 2008): on stiff substrates, the cellular traction forces quickly break new adhesions, leading to a slip regime. On soft substrates, the cells can pull back while building up traction forces, gradually increasing load on the adhesions until they fail (Chan and Odde, 2008). Coupling multiple motor-clutch units, cell anisotropy emerges with an optimum that depends on the traction force and traction rate as well as on the number of motor-clutch units (Bangasser et al., 2017). Also, durotaxis emerges at a single cell (Bangasser et al., 2017) or collective (Sunyer et al., 2016) cell level.

Altogether, previous models suggest that mechanosensing is regulated by positive feedback between substrate stiffness, traction force, and/or FA stability. These models included many feedback mechanisms, with detailed dynamics of cytoskeleton/F-actin (Deshpande et al., 2008; Vernerey and Farsad, 2014), adhesion, and/or thermodynamics principles (McEvoy et al., 2018; Shishvan et al., 2018), resulting in a multiplicity of variables and parameters. On the plus side, such detailed models can be used to fit specific experimental data well and to incorporate facts from the experimental literature. On the minus side it remains unclear how the different mechanisms work together, which ones are essential or redundant, and what their respective roles are in distinct cell behavior such as elongation versus uniform spreading. Can durotaxis emerge from the same set of assumptions, or are additional mechanisms required?

In many models, it is assumed that cells become more contractile on stiffer substrates. However, it has been shown in fibroblasts that contractile forces do not depend on substrate stiffness (Feld et al., 2020; Freyman et al., 2002). Here we show that mechanosensitive focal-adhesion turnover, together with cell contractility and substrate adhesion suffice to explain all three ECM-stiffness-dependent cell behaviors. Our model suggests that cells distinguish between soft and stiff substrates not by adjusting the force magnitude, but rather the rate of force buildup: forces on FAs increase much faster on stiff substrates than on soft ones.

The model is based on the following assumptions: **(1)** The ECM is a nonlinear elastic material, such as a polyacrylamide gel. **(2)** FAs are discrete clusters of integrin-ECM bonds. **(3)** New bonds are added to the FAs from a pool of available free integrin bonds on the cell membrane. We assume a constant rate once the first adhesion bonds bring the cell membrane close enough to the ECM (Novikova and Storm, 2013; Sun et al., 2009, 2011). **(4)** The unbinding rate is suppressed by the tension in the FA due to pulling of stress fibers (Novikova and Storm, 2013). **(5)** On soft ECMs it takes more time for the tension in the FA to build up to its maximal value, than on stiff ECMs (Schwarz et al., 2006). **(6)** Larger FAs detach less easily from the ECM. **(7)** Planar stress reinforces FAs due to recruitment of stabilizing proteins such as vinculin.

As we will show, the model predicts that the range of stiffness on which cells elongate depends on the velocity of myosin motor proteins. We find that simulated cells exhibit durotaxis. Consistent with experimental observations, the durotaxis speed increases with the slope of the stiffness gradient (Isenberg et al., 2009; Vincent et al., 2013). Because the key to our proposed model is mechanosensitivity of the FAs, it is likely generalizable to nonlinearly elastic, inhomogeneous, and fibrous natural matrices.

## Results

### Modeling Approach and Details

**Figure 1:**
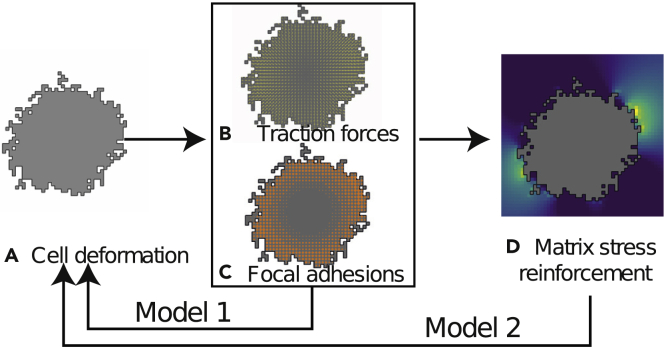
Flowchart of the Model (A–C) In Model 1, (A) the cellular Potts model (CPM) predicts cell deformation based on contractile forces due to surface tension, balanced by cell-ECM adhesion and activity of the FAs; (B) additional cellular traction forces are calculated based on cytoskeleton bulk contractility and matrix stiffness; (C) the build-up of traction forces is calculated jointly with the growth of the FAs. (D) In Model 2, the stability of the FAs also depends on planar stress in the ECM, calculated using a finite-element model.

We have extended our hybrid Cellular Potts—Finite Element framework (van Oers et al., 2014; Rens and Merks, 2017) to include dynamic descriptions of FAs. Figure 1 gives a flowchart of the model, showing the feedback between the cell, the FAs, and the elastic substrate it adheres to (see the Transparent Methods for details).

#### Cells

A cell is described as a set of discrete lattice sites in a cellular Potts model (CPM) (see Figure 1A). Cells in the CPM change shape by iteratively making extensions and retractions of the cell boundary to adjacent lattices sites. This process also accounts for the formation and degradation of adhesions with the substrate. Time is measured in so-called Monte Carlo Steps (MCS), during which all sites along the cell boundary are given the opportunity to move. An additional rule is introduced to model the adhesive effect of FAs (assumption **(6)**): an energy barrier proportional to the size of the FA reduces the probability that the cell retracts from the substrate.

#### Cell Traction Forces

In addition to the retraction forces given by the CPM, we assume that the cell applies contractile forces onto each of the FAs. Based on the shape of the cell, we calculate the contractile force using the First Moment of Area (FMA) model (Lemmon and Romer, 2010) (Figure 1B). This model assumes that the cell acts as a single contractile unit, resulting in a force onto each of the FAs pointing toward the center of mass and proportional to the distance to the center of mass. The forces at the individual FAs build up slowly from the latest force applied on the FA during the last MCS, to the force given by the FMA model at the given site and current MCS. For the latter, we use a model previously proposed by Schwarz et al. (2006) in which the force builds up faster on stiffer substrates (assumption **(5)**).

#### Focal Adhesions

We model FAs as clusters of catch-slip bonds (assumption **(2)**), as proposed by Novikova and Storm (Novikova and Storm, 2013). This model is based on experimental data of a single α5-β1 integrin that has constant binding rate (assumption **(3)**) and assumes that the degradation rate of bound integrins decreases with force (assumption **(4)**). The assembly and disassembly of a FA is then given by the collective dynamics of a cluster of integrin bonds. At each site of our 2D CPM, we implement the dynamics of one such FA (Figure 1C). Note that the FAs vanish near the cell center, because of insufficient traction. To increase visibility in the next figures, we visualize every other FA and show the average FA size of the surrounding four neighboring lattice sites.

#### Simulation

The simulations proceed as follows. In **Model 1**, we initiate cells in the CPM (Figure 1A). Then, we let the forces build up for *t*_FA_ seconds (Figure 1B) and let the FAs grow simultaneously (Figure 1C). After that, we let the cells move for one MCS (Figure 1A) and repeat. **Model 2** includes an extra step in which planar stress in the linear elastic ECM substrate (assumption **(1)**) reinforces the FAs (assumption **(7)**) (Model 2.1) or increases the forces applied to them (Model 2.2) (Figure 1D).

#### Parameter Values

Where possible, parameter values were chosen based on literature values (Table S1), or else estimated, followed by parameter sensitivity studies.

### Model Predictions

#### Catch-Bond Cluster Dynamics Suffices to Predict Cell Area as a Function of Substrate Stiffness

**Figure 2:**
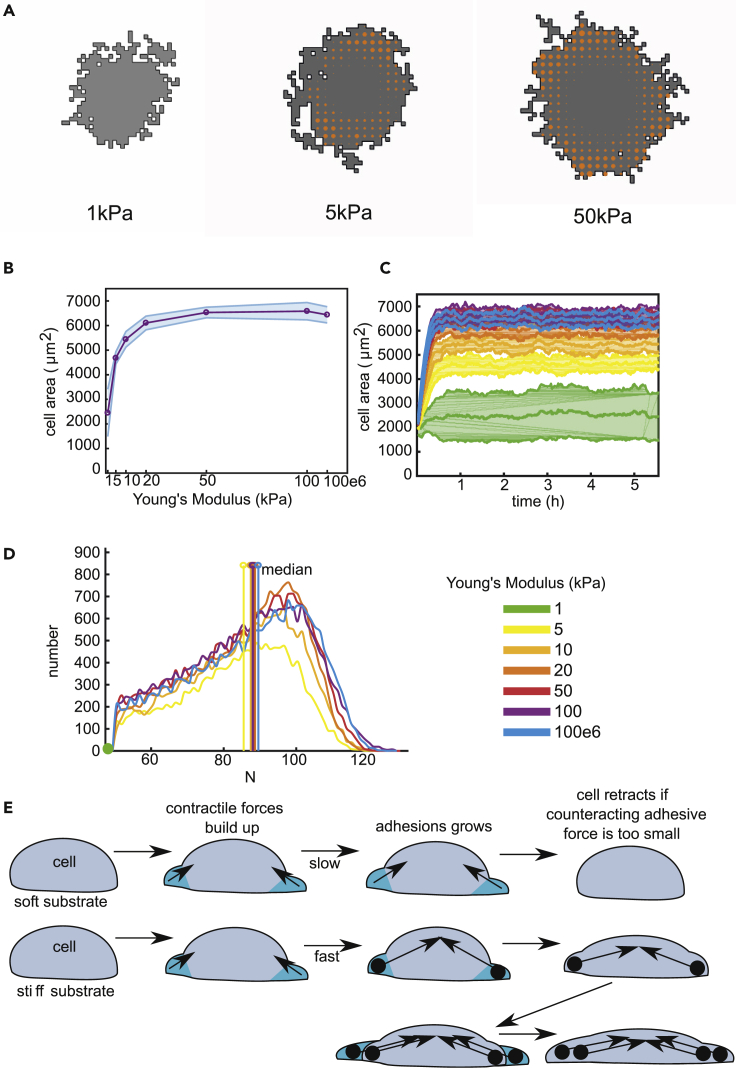
Model 1 Predictions: Cell Area Increases with Increasing Substrate Stiffness (A) Example configurations of cells at 2000 MCS on substrates of 1, 50, and 50 kPa. (B) Cell area as a function of substrate stiffness, shaded regions: standard deviations over 25 simulations. (C and D) (C) Time-series of cell area, shaded regions: standard deviations over 25 simulations; and (D) distribution of N, the number of integrin bonds per cluster, all clusters at 2000 MCS from 25 simulations were pooled. We indicate the median. Color coding (C and D): see legend next to (D). (E) A cartoon to schematically explain spreading of a cell based on our model. Top: cell on a soft substrate; bottom: cell on a stiff substrate. The cell forms protrusions (cyan) that either successfully get stuck to the ECM or are retracted. The cell starts to build up a force, which builds up much faster on stiff substrate. Thus, on stiff substrates, FAs stabilize. Because the counteracting adhesive force is too small, the cell retracts from the soft substrate, whereas the cell is able to stick and spread further on a stiff substrate. See also Video S1, Table S2, Figures S1, S4A, and S5.

We first tested whether the build-up of stiffness-dependent forces alone suffices to explain the observed cell deformation. We simulated Model 1 on a substrate of size 500 *μ*m by 500 *μ*m with stiffness of 1kPa, 5kPa, and 50kPa for 2000 MCS (≈5.5 h) (Figure 2A and Video S1). On the softest substrate of 1kPa, FAs did not grow and the cell did not spread. On a slightly stiffer substrate of 5 kPa, FAs grew and the cell increased in area. On the stiffest substrate of 50 kPa, the cell area further increased. Large FAs became visible around the cell perimeter (Figure 2A). The cell area increased 2.5-fold (2500 *μ*m^2^–6500 *μ*m^2^) as stiffness increased up to 50 kPa (Figure 2B), consistent with experimental observations (Asano et al., 2017; Balcioglu et al., 2015; Califano and Reinhart-King, 2010). We compared our model predictions with empirical functions relating substrate stiffness to cell area proposed in Balcioglu et al. (2015) and in Engler et al. (2004) (Figures S1B-D) and found these to be consistent, apart from incidental quantitative discrepancies (See Table S2).

Video S1 Cell Spreading on Substrates of 1,5 and 50kPa (Model 1), Related to Figure 2 These are time series of Figure 2A of 500 MCS.

Comparing predicted and observed spreading kinetics (Figure 2C), we found qualitative resemblance for endothelial cells (Reinhart-King et al., 2005; Yeung et al., 2005) and fibroblasts (Nisenholz et al., 2014; Yeung et al., 2005). Cells spread to their final size over 30 min, a timescale consistent with fibroblasts (Yeung et al., 2005), but somewhat shorter than endothelial cells *in vitro* (100 min (Reinhart-King et al., 2005)). To compare the timescale of spreading, we consider *t*_50_: the time for a cell to reach 50% of its maximal area. To estimate *t*_50_, we fit an error function $A\left(t\right)=A_{50}\cdot \left(1+erf\left(\frac{t-t_{50}}{\tau }\right)\right)$ (Reinhart-King et al., 2005) to the simulated curves A(t). In our simulations, we found that $t_{50}\approx 5$ min (with 95% confidence interval of [5.02,5.46]) for cells on a 50kPa substrate (Figure S1K). In Reinhart-King et al. (2005), $t_{50}\approx 50$ min for BAECs on RGD-derivatized PA gel. The model parameter $t_{\text{FA}}$ sets the time scale of FA maturation (time for FA growth before the cell can extend or retract). Higher values of $t_{\text{FA}}$ result in slower cell spreading (Figure S5A).

We next analyzed the dependence of final cell area on substrate stiffness for a range of values of $t_{\text{FA}}$ (Figure S5B). Because cell spreading and stiffness were most strongly correlated at $t_{\text{FA}}=10\;s$ (slightly less than the lifetime of cellular protrusions (Knorr et al., 2011)), we used this as a default value for other simulations. Figures S5C–F shows similar sensitivity analyses for the cell spreading parameter, $\lambda_{C}$, and the FA growth rate, *γ* (see Transparent Methods). Thus, we could obtain spreading kinetics resembling those of endothelial cells (Reinhart-King et al., 2005) by increasing FA maturation time and reducing FA growth rate (Figures S5G–H).

For human fibroblasts (Nisenholz et al., 2014), the timescale of spreading, *τ*, decreases as the stiffness of the substrate increases (Figure S1E). Fitting the inverse exponential function (Figure S1J) from (Nisenholz et al., 2014), we obtain a similar dependence of *τ* on stiffness (Figure S1H). As shown in Figure S1J, our fit is 4 times faster, but, as previously shown (Figures S5G and S5H), varying free parameters can produce even closer agreement. In Nisenholz et al. (2014), the rate of change of the area ($\frac{dA}{dt}\left(t\right)$) was shown to initially increase before dropping down to 0 (Figure S1G). On the stiffest substrate, $\frac{dA}{dt}\left(t\right)$ was overall much faster (Figure S1G). Interestingly, even without explicitly putting such dynamics into our model, a very similar curve emerges (Figure S1I).

We next investigated the distribution of the FA sizes (Figure 2D). At 1kPa, all FAs did not grow above the nascent adhesion threshold ($N_{0}=50$), so they were assumed to break apart (see Transparent Methods). The median cluster size and variance were unaffected by substrate stiffness, in contrast with Prager-Khoutorsky et al. (2011) and Trichet et al., 2012 who observed larger adhesions on stiffer substrates. Interestingly, and in line with our model observations, Balcioglu et al. (2015) report that average adhesion size is independent of substrate stiffness, although cell area and number of adhesions increase. A more detailed analysis of the distribution of the FAs revealed that stiffer substrates enhance the proportion of larger FAs, as observed in Paszek et al. (2005) (20% on 50,000 kPa, 15% on 10 kPa, and 10% on 5 kPa for FA with N>100). Furthermore, on stiffer substrates, large FAs were found not far from the cell periphery (Figure 2A for 50 kPa and Figure S1). Similarly, in Shemesh et al. (2012) and in Shemesh et al. (2009) FAs were observed at the lamellipodium-lamellum interface, a small distance away from the cell edge. Choi et al. (Choi et al., 2008) attributed this observation to slow integrin-actin association, but in our model, this emerges from the fact that cell interior forces have more time to develop, thus stabilizing adhesions. On softer substrates, the largest FAs are found closer to the cell center (Figures 2A and S1). Close to the cell edge, cells frequently create and destroy adhesions by protrusion/retraction. Experimental images in Prager-Khoutorsky et al. (2011), Balcioglu et al. (2015), and Wormer et al., 2014 appear to demonstrate that FAs grow further from the cell edge on softer substrates.

In the present model, we have assumed that the ECM can be approximated as a homogeneous, linearly elastic material. Although this can be a suitable approximation for small deformations of synthetic matrices, such as functionalized polyacrylamide matrices (Storm et al., 2005), natural matrices are highly inhomogeneous and nonlinearly elastic. To assess the effect of local inhomogeneities of ECM stiffness or of fluctuations in stiffness measurements by the cells (Beroz et al., 2017), we next simulated ECM inhomogeneity by applying random spatial variations to the Young's modulus of the ECM (Figure S4A). Interestingly, the degree of inhomogeneity had little effect on the cell spreading area, because spreading is due to the average behavior of the FAs in the whole cell.

##### Mechanism of Uniform Cell Spreading

All in all, the mechanosensitive kinetics of the FAs suffices to predict stiffness-dependent cell area and spreading dynamics in a noise-resistant way. A schematic overview of our proposed mechanism for uniform cell spreading on soft versus stiff substrates is shown in Figure 2E. Cells (gray) send out protrusions (cyan) to probe the substrate by gradually building up forces. On a soft substrate, forces develop slowly, and thus adhesions do not grow enough to stick the cell to the substrate, and the cell retracts its protrusion. On a stiff substrate, forces build up rapidly, causing FAs to stabilize so the cell adheres to the substrate. This process repeats with new protrusions, allowing for maximal cell spreading.

#### Focal Adhesion Strengthening due to Matrix Stress Induces Cell Elongation

Model 1 correctly predicts the effect of substrate stiffness on cell spreading, but it cannot yet explain cell elongation. As cells pull, they deform the ECM, generating planar stresses. Uniaxial stretching of the substrate speeds up FA assembly, leading to FA strengthening and cell elongation, whereas on a longer timescale, planar stress induces FA disassembly, dampening cell elongation (Chen et al., 2013). We therefore hypothesize that an effect of planar stress on FAs may be involved in stiffness-induced cell elongation.

##### Model 2.1: Planar Stress Reinforces FAs

To predict the planar stress resulting from cellular traction forces, we extended Model 1 with a finite-element model of the substrate (Figure 1D) (van Oers et al., 2014; Rens and Merks, 2017). To model the effect of planar stress on FA reinforcement, we multiplied the energy to detach an FA from the substrate by a factor of $1+p\frac{g\left(\underline{\sigma }\left(\vec{x}\right)\right)}{\sigma_{h}+g\left(\underline{\sigma }\left(\vec{x}\right)\right)}$. The parameter *p* regulates the strength of this effect, $\sigma_{h}$ is a saturation parameter, and $g\left(\underline{\sigma }\left(\vec{x}\text{'}\right)\right)$ gives the hydrostatic stress on the FA.

**Figure 3:**
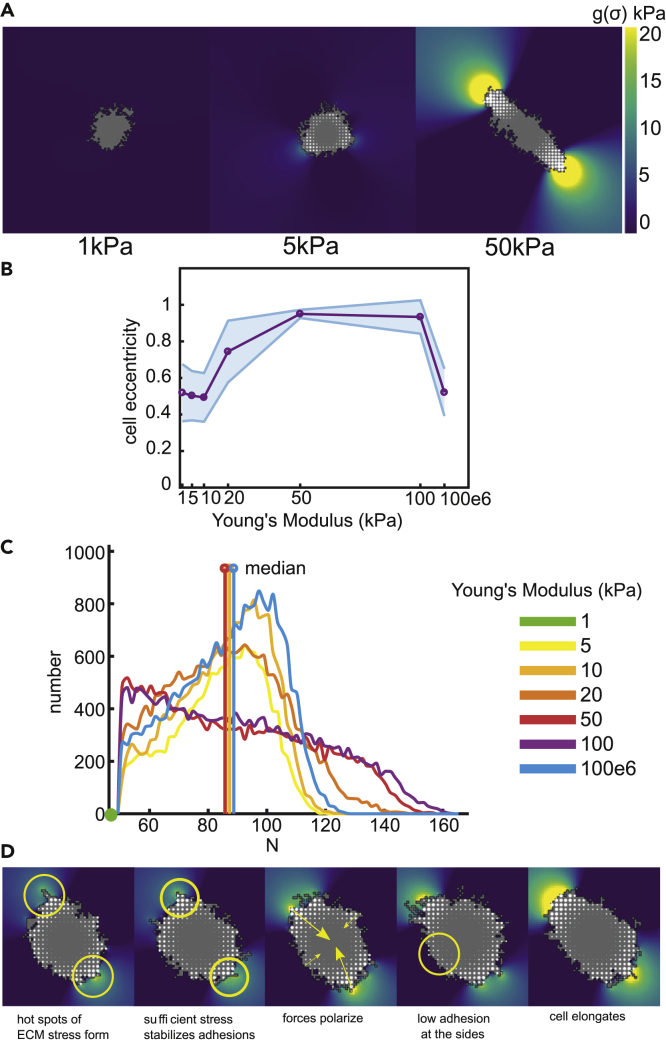
Cells Elongate on Substrates of Intermediate Stiffness (A) Example configurations of cells at 2000 MCS on substrates of 1, 50, and 50 kPa. Color ramp shows hydrostatic stress. (B and C) (B) Cell eccentricity as a function of substrate stiffness, shaded regions: standard deviations over 25 simulations; (C) distribution of N, the number of integrin bonds per cluster, all FA at 2000 MCS from 25 simulations were pooled. Vertical line piece shows the median value of the FA sizes. Color coding for panel (C): see legend. (D) The mechanism of cell elongation explained. Hot spots of ECM stress form under protrusions. If this stress is strong enough, the FA stabilizes here, obstructing cell retractions. Around these hot spots, forces will continue to build up and the force field polarizes. FAs continue to stabilize at the front and back, whereas adhesions at the sides of the cell remain small. This feedback loop allows the cell to elongate. See also Videos S2 and S3, Figures S2, S3, S4, and S6.

Figure 3 (Video S2) shows typical cell configurations resulting from Model 2.1. As in Model 1, cells stay small and round on the softest substrate (1 kPa), elongate somewhat on stiffness of 20 kPa, and elongate significantly on stiffer substrates (50–100 kPa). Figure 3B shows the eccentricity of cells ($\sqrt{1-{\left(b/a\right)}^{2}}$, with *a* and *b* the semi major and minor axes). On 50–100 kPa matrices, large FAs form at the two poles of the cell. On even more rigid substrates, the cells return to a circular shape. The same biphasic dependence of cell eccentricity on substrate stiffness was also experimentally observed for hMCS cells elongating most strongly on substrates of 10 kPa (Zemel et al., 2010). In general, the substrate rigidity associated with maximal elongation is cell-type and matrix-composition dependent. But since only a small range of substrate stiffness is usually tested, information is lacking about the exact stiffness at which cells start to elongate. For example, fibroblasts do so at 2kPa on collagen-coated PA gels. On fibronectin-coated PA gels, fibroblasts with PTK knockdown failed to elongate strongly at 30kPa but did so at 150kPa (Prager-Khoutorsky et al., 2011). MCS elongated at 9kPa (but not at 0.7kPa (Rowlands et al., 2008)), endothelial cells at 1kPa (Califano and Reinhart-King, 2010), and cardiomyocytes at 5kPa (Chopra et al., 2011).

Video S2 Cell Spreading on Substrates of 1,5 and 50kPa (Model 2.1), Related to Figure 3 These are time series of Figure 3A of 500 MCS.

We next tested how local inhomogeneities in substrate stiffness affect the ability of cells to elongate. As before, we added uniform noise to the substrate stiffness. Figure S4B shows the cell eccentricity as a function of ECM stiffness and the level of inhomogeneity; together these data suggest that the observed cell elongation is robust to noise. Next, we investigated the effect of inhomogeneities on a longer length scale, by imposing a sinusoidal function of substrate stiffness and varying its period and amplitude (see Transparent Methods and Figures S4D-L). Cells elongate as before, unless the period of the sinusoid is much greater than the cell diameter and the stiffness significantly deviates from its baseline (50kPa, Figures S4K-L). This suggests that if the ECM stiffness changes significantly on a length scale of a cell, the cell cannot find two stiff-enough anchor spots so as to elongate.

We compared the distribution of FAs of elongated cells to uniform cell spreading. Figure 3C shows the distribution of the FA sizes as a function of substrate stiffness. As before, the median cluster size does not vary much with substrate stiffness. The shape of the distributions, however, are flatter, with higher variance for elongated cells compared with round cells (kurtosis k≈2.0, SD ≈2700 for elongated cells and k≈2.3, SD ≈1500 for round cells). This larger variation in FAs sizes in elongated cells stems from the polarized force field that forms large FAs at the poles and small FAs at the lateral sides.

We next performed a sensitivity analysis for parameters not constrained by experimental data. For increased values of *p*, which regulates planar stress-induced FA strengthening, cells elongate over a much wider range of stiffnesses (Figures S2A-B). The value of the saturation parameter $\sigma_{h}$ has little effect on the behavior of the model (Figures S2C-D). Other parameters that might reflect cell-type-dependent differences include the lifetime of the protrusions $t_{\text{FA}}$ (Figures S2E-F), the cellular motility parameter *T* (Figures S2G-H), and the magnitude of the traction forces *μ* (Figures S2I-J). The qualitative behavior does not depend on the values of these parameters, but they do affect the range of substrate stiffness on which cells elongate.

Because of the relatively large cellular temperature *T*, the cell contours are fairly rugged. This temperature value was required to stabilize the elongated cell shapes. Although a lower temperature can still produce similar behavior (Figure S6A), we observed that elongated cells go through cycles of collapse (when contractile force is higher than adhesive force) and elongation. Higher temperature ensures that cells are sufficiently motile, rapidly forming new protrusions and adhesions after a slight retraction.

##### Model 2.2: Planar Stress Enhances Traction Force

We also tested an alternative model in which the force exerted on the FAs increases in response to planar stress. We assumed that the stall force increases as a function of matrix stress, i.e., ${\vec{F}}_{s}={\vec{F}}_{s}\left(1+p\frac{g\left(\underline{\sigma }\left(\vec{x}\right)\right)}{\sigma_{h}+g\left(\underline{\sigma }\left(\vec{x}\right)\right)}\right)$. This alternative mechanism produces ECM-stiffness-dependent cell morphologies that are similar to the default model (Figure S3 and Video S3). This alternative mechanism could have various molecular origins. For instance, addition of vinculin through talin stretching can induce increased traction forces (Dumbauld et al., 2013). Stretching forces also induce α-smooth muscle actin recruitment to stress fibers (Goffin et al., 2006) and myosin motor binding (Uyeda et al., 2011).

Video S3 Cell Spreading on Substrates of 1,5 and 50kPa (Model 2.2), Related to Figure S3 These are time series of Figure S7A of 500 MCS.

##### Mechanism of Cell Elongation

In conclusion, our model suggests a mechanism for cell elongation that we illustrate with a series of snapshots in Figure 3D. Because of stochastic variations, slightly eccentric cell shapes are formed occasionally. The first-moment-of-area (FMA) model (Lemmon and Romer, 2010) predicts that the cell generates high forces around protrusions, forming local hot-spots of ECM stress (yellow circles in Figure 3D1,2) in the underlying substrate. Because we assumed that ECM stress stabilizes FAs (Model 2.1), protrusions around such hot-spots stick to the ECM. Then, forces can further develop, ECM stress increases, and FAs further stabilize. As the cell elongates, the force field also polarizes (Figure 3D3). On the lateral sides of the cell, traction forces are smaller and thus insufficient to maintain stable FAs (Figure 3D4). Together with the positive feedback between stress and FAs, the retractions at the lateral sides reinforce cell elongation on substrates of intermediate stiffness. In version 2.2 of our model, cell elongation happens similarly, but here ECM stress positively feeds back on cell traction forces, rather than on the adhesive force. On soft substrates, force build-up is not strong enough to stabilize the FAs, whereas on stiff substrates, all FAs stabilize, leading to round cells on both soft and stiff substrates.

##### Contractility Rate Changes Stiffness Regime on which Cells Elongate

**Figure 4:**
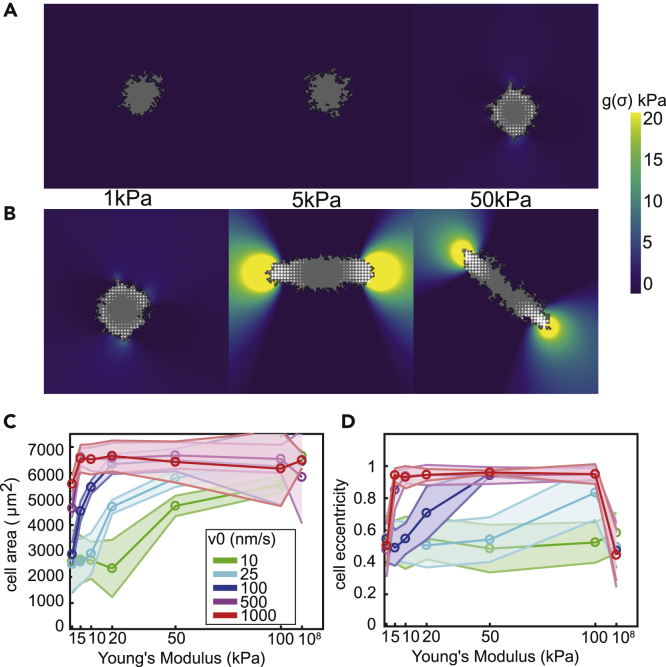
The Range of Stiffness on which Cells Elongate Depends on Myosin Motor Velocity Model 2.1 was used. (A) Example configurations of cells at 2000 MCS on substrates of 1, 50, and 50 kPa with motor velocity 10 nm/s. (B) Example configurations of cells at 2000 MCS on substrates of 1, 50, and 50 kPa with motor velocity 1,000 nm/s. Colors (A and B): hydrostatic stress; (C) mean cell area as a function of motor velocity, error bars: standard deviations over 25 simulations; (D) mean cell eccentricity as a function of motor velocity, error bars: standard deviations over 25 simulations.

There are large differences in the response to ECM stiffness between cell types. Fibroblasts spread out further on stiff ECMs than on soft ECMs, whereas endothelial cells do so to a lesser extent (Yeung et al., 2005). Cells display a large variability in contractility (Murrell et al., 2015) and in the actomyosin contraction rate, which may be suppressed by microtubules (Bershadsky et al., 2003a; Dugina et al., 2016). To test whether variability in the response to ECM stiffness could be due to differences in the rate of actomyosin fiber contraction, we varied cell contractility by modulating the velocity of the myosin motors, $v_{0}$, during force build-up (see Equation 5 in Transparent Methods). We studied a range from 10 nm/s, corresponding to nonmuscle myosin-IIB (Norstrom et al., 2010) to 1,000 nm/s, corresponding to muscle myosin (Vogel et al., 2013). Figures 4A and 4B show the cell configurations for reduced ($v_{0}=10\;\text{nm}/\text{s}$) and increased motor velocities ($v_{0}=1000\;\text{nm}/\text{s}$), corresponding to velocities one order of magnitude below and above the default value ($v_{0}=100\;\text{nm}/\text{s}$). Figures 4C and 4D plot the cell area and cell eccentricity as a function of motor protein velocity averaged over 25 independent simulations. With reduced motor velocity, the cells spread only on the stiffest matrices tested (Figure 4C) and failed to elongate (Figure 4D). Cells with increased motor velocities, however, elongated at relatively soft matrices of 5 kPa (Figures 4B and 4D).

#### Durotaxis Explained by a Bias in FA-Turnover Rate

We next asked whether our model mechanism also suffices to predict durotaxis: cell migration up a stiffness gradient (Lo et al., 2000). It has previously been proposed that cells durotact due to increased FA maturation on stiff parts of the matrix, whereas the FAs disintegrate and cellular protrusion retract at softer parts of the substrate (Wong et al., 2014). We tested this in-silico by placing cells on stiffness gradients and following their movement for 10,000 MCS (≈28h). The stiffness increased linearly over the x axis from 1 kPa (left) to 26 kPa (right), corresponding to a slope of 20 Pa/*μ*m. The cell starts at (250μm,250μm) at 6 kPa. We found that cells gradually drifted toward the right, the stiffer part of the gradient on homogeneous matrices (Video S4).

**Figure 5:**
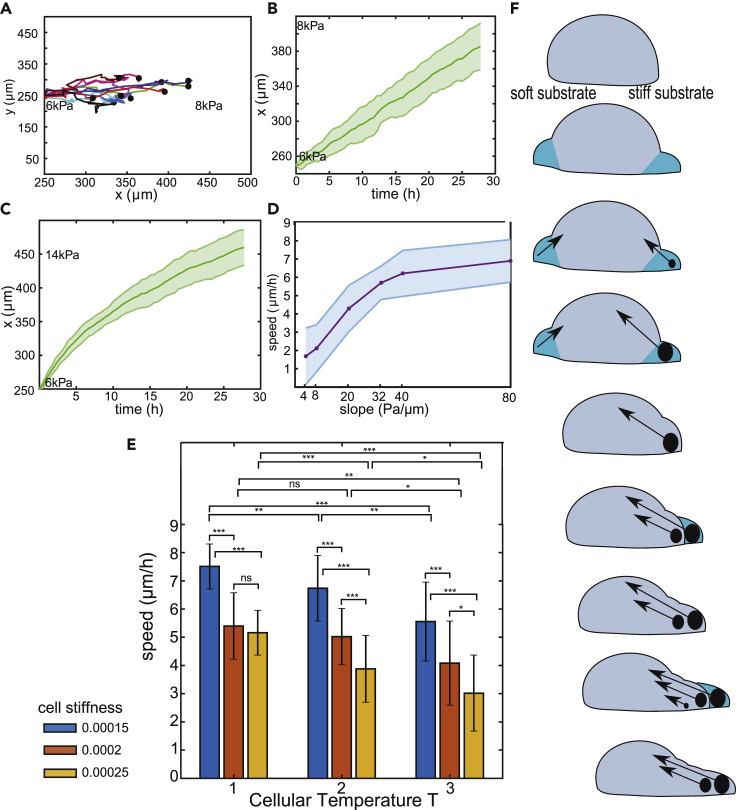
Durotaxis as a Result of Integrin Catch-Bond Dynamics (A) Ten trajectories of durotacting cells on a matrix with slope 20 kPa/*μ*m. (B) X-coordinate of the cell as a function of time, on a matrix with slope 20 kPa/*μ*m. (C) X-coordinate of the cell as a function of time, on a matrix with slope 80 kPa/*μ*m. (D) Cell speed as a function of the slope of the stiffness gradient. (E) Durotaxis speed in *μ*m/h as a function of cell stiffness *λ* and cellular temperature *T*. Values: mean ± standard deviation over 25 simulations. Comparisons indicated by asterisks (∗∗∗p<0.001, ∗∗p<0.01, ∗p<0.05, ns: nonsignificant, i.e., p>0.05) based on ANOVA followed by Student's t test. (F) A cartoon to schematically explain durotaxis based on our model. The cell forms protrusions (cyan) that either successfully get stuck to the ECM or are retracted. The cell experiences a stiffer substrate on the right, so forces develop much faster on the right, allowing FAs to stabilize there. At the left, FAs fail to stabilize, allowing the cell to retract in order to propel itself forward and generate new FAs at the front. This continues and the cell moves to the right. See also Video S4, Figure S4C.

Video S4 Cell Durotaxis on a Substrate with Rigidity Gradient 20 Pa/μm, Related to Figure 5 Time series of 14000 MCS.

The simulated cells move in the *x*-direction at constant velocity (Figure 5B), with an average speed of 4.3μm/h, (average slope, dx/dt, over 25 simulations), comparable to 6.2 *μ*m/h for mesenchymal stem cells on a stiffness gradient of 20 Pa/*μ*m *in vitro* (Vincent et al., 2013). In DuChez et al. (2019), human cancer cells moved faster on stiffer substrate, whereas, in contrast, our simulations predict slower cell motion when cells reach a stiffer substrate (Figure 5C). Interestingly, when human cancer cells were placed on a steeper gradient, cell movement did not depend on substrate stiffness: cells neither sped up nor slowed down (DuChez et al., 2019). In our model, slowing on stiff substrates results from saturated growth of FAs: cells hardly sense a left-right difference on a stiff substrate. Actual cells may speed-up as they polarize due to signaling between ECM contacts and GTPases Rac and Rho (Park et al., 2017). If durotaxis depends on front-rear differences in FA, as suggested in Wong et al., 2014, one would expect faster cell motion on steeper stiffness gradients, where the difference in FA stability should be greater. Indeed, as shown in Figure 5D, simulated cells move faster on steeper gradients, as observed experimentally for vascular smooth muscle cells and mesenchymal cells (Isenberg et al., 2009; Vincent et al., 2013). This observation depends on cell type: human cancer cells were shown to move less persistently on steeper stiffness gradients (DuChez et al., 2019). This discrepancy with Vincent et al. (2013) and Isenberg et al. (2009) might have to do with cytoskeletal changes in cancer cells.

##### Mechanism of Durotaxis

We illustrate our proposed mechanism for durotaxis in Figure 5F. The cell probes the substrate by randomly sending out protrusions. Because forces build up faster on the stiff side of the substrate, FAs grow larger than on the soft side. Protrusions are thus more likely to stick to the stiff part, allowing the cell to move forward as it retracts its back and new protrusions successfully stick in front. We tested the role of the feedback between ECM stress and FA stabilization, by setting p=0, equivalent to running Model 1. Interestingly, cells durotact (but slightly slower, at 3.9μm/h), suggesting that the catch-bond integrin dynamics are sufficient for durotaxis and any further stabilization of FA proteins makes cells move quicker.

The durotaxis mechanism requires that cells are sufficiently flexible to make new protrusions and retract old ones. Indeed, increasing the value of *λ* that decreases cell flexibility (See Equation 3 in Transparent Methods) reduces cell speed (Figure 5E). Because increasing *λ* restricts protrusions, this could be associated with inhibiting actin polymerization. Similar to our prediction, cells treated with cytochalasin D, an actin-polymerization inhibitor, failed to spread and durotact (Vincent et al., 2013). Furthermore, inhibition of actin dendritic nucleation inhibits durotaxis (DuChez et al., 2019).

Durotaxis speed should also depend on how reliably matrix stiffness controls the rate of protrusions and retractions. In the CPM, the stochasticity of such shape changes is controlled by the “cellular temperature'' *T* (See Equation 2 in Transparent Methods), where for larger values of *T*, retractions are less tightly controlled by cell-substrate adhesion and other environmental and cellular properties. As predicted, cell speed is reduced for larger values of the cell motility parameter (Figure 5C). We also tested to what extent matrix inhomogeneity affects the durotactic migration speed of cells; even noise levels of ±10kPA did not affect the durotactic cell velocity (Figure S4C).

## Discussion

We have shown that mechanoregulation of the assembly and disassembly of FAs by matrix stiffness and planar stress suffices to explain (a) increased cell spreading on stiff substrates (Figures 2A-B), (b) cell elongation on substrates of intermediate stiffness (Figures 3A-B), and (c) durotaxis (Figure 5A). Many previous mathematical models of cell spreading and cell migration used a bulk equation for cell-matrix adhesion, motivated by the underlying FA dynamics (Bischofs and Schwarz, 2003; Kabaso et al., 2011; Ni and Chiang, 2007; van Oers et al., 2014; Ramos et al., 2018; Zemel et al., 2010). More closely related to the present work, hybrid cell and FA models were developed to study how the mechanosensitive growth of FAs direct cell spreading and migration (see, e.g., Copos et al. (2017) and Uatay (2018)). It was proposed that cell spreading is regulated by a matrix-stiffness-induced stress fiber persistence and upregulated traction forces (Ronan et al., 2014; Vernerey and Farsad, 2014). Another model has proposed an enhanced cell contraction on stiff matrices (Stolarska and Rammohan, 2017). Because this effect counteracted cell spreading, the model could not explain increased cell spreading on stiff matrices. These previous models suggest that an upregulation of cell forces is required to induce cell spreading on stiff matrices. Similarly, Novikova and Storm (Novikova and Storm, 2013) noted that force evolution alone does not enable a cell to distinguish soft from stiff matrices, because ultimately the same stall force is reached. They propose that stiffness sensing requires cells to apply more force on stiffer substrates. Our model suggests that force evolution can be sufficient for cells to sense matrix stiffness. In our model, cells can more rapidly build up forces on stiffer matrices during the lifetime of a cell protrusion, allowing FAs to stabilize and protrusions to stick to the matrix and subsequently the cell to spread. On soft matrices, the force does not reach a sufficient level during the lifetime of a protrusion to allow for stabilization. So, force evolution together with the dynamic nature of protrusions/retractions enable the cell to distinguish stiff from soft.

For uniform cell spreading, it is essential that (a) FAs apply an adhesive force on the cell (clearly the case, based on the known function of FAs), and (b) the rate of force build-up depends on ECM stiffness. Otherwise, cells are not able to distinguish between soft and stiff matrices and spread accordingly. After adding the assumption that planar stress contributes to the stabilization of FAs, the predictions of our model also sufficed for cell elongation. A possible molecular mechanism for this effect is the stretching of talin in FAs. Stretching of talin exposes vinculin-binding sites (Rio et al., 2009). Vinculin in turn binds the FA to the cytoskeleton, which strengthens cell-matrix adhesion (Gallant et al., 2005). In agreement with this hypothesis, vinculin regulates cell elongation on glass substrates (Ezzel et al., 1997). Although observations in fibroblasts suggest that traction forces are independent of ECM stiffness (Feld et al., 2020; Freyman et al., 2002), as we assumed in this model, there are also contradicting observations: (1) vinculin increases cell traction forces (Dumbauld et al., 2013), and (2) stressing FAs induces α-smooth muscle actin recruitment to stress fibers, which in turn increases traction forces (Goffin et al., 2006). We have, therefore, also tested an alternative mechanism in which planar stress induces an increase in cell traction forces (Figure S3); this mechanism also suffices for cell elongation. Future, quantitative comparisons of our model predictions and *in vitro* observations may help elucidate which of these two mechanisms, if any, best explains cell elongation and for what cell types.

Our model also predicts distinct ranges of substrate stiffness for cell elongation could be due to diverse myosin motor velocities (Figure 4), possibly accounting for cell-type dependence (Georges and Janmey, 2005; Yeung et al., 2005). For example, in ovarian tumor cells deficient in Dlc1 (responsible for phosphorylation of nonmuscle IIA myosin (Sabbir et al., 2016)), elongation is promoted, suggesting that an increase in motor velocity indeed facilitates greater cell elongation. This prediction is experimentally testable by studying cells that express different isoforms of myosin or by overexpression or inhibition of a given myosin isoform in cells while systematically varying substrate stiffness.

The assumptions of our model also sufficed for durotaxis. The mechanoresponsivity makes FAs longer-lived on the stiffer side of the matrix than on the softer side of the matrix. The resulting bias in FA turnover and pseudopod turnover is responsible for a drift of the cell toward the stiffer side of the matrix. Instead of such implicit, emergent effects, previous models of durotaxis have often assumed direct effects of ECM stiffness on FA density and polarity (Kim et al., 2018; Yu et al., 2017). Feng et al. (Feng et al., 2018) showed that if FA degradation is higher in the back than in the front *and* FAs mature under applied force, then a cell can durotact. Based on experimental observations, Novikova et al. assumed that cells move more persistently on stiffer substrates and showed that a persistent random walk can reproduce durotaxis (Novikova et al., 2017). In contrast to the direct effect of ECM stiffness on cell polarization, in our model durotaxis emerges from a bias in FA turnover. By knocking-out the model feedback between ECM stress and FA stabilization (obtaining Model 1), we found that durotaxis is still possible, but at a slightly reduced speed. This suggests that the catch-bond integrin behavior suffices for durotaxis. The role of vinculin could then be to increase cell speed. This prediction can be tested empirically by knocking down vinculin or related structural proteins.

Our model predicts that durotaxis speed saturates with steeper stiffness gradients. In our model, this emerges from the fact that the growth of FAs saturates at high ECM stiffness, giving an upper bound to the cell speed. To validate this prediction, a wider range of stiffness gradients should be investigated. Available data so far only include a limited range of stiffness gradients (Isenberg et al., 2009; Vincent et al., 2013), suggesting possibly interesting future experiments.

### Model Limitations

No model can account for every experimental observation and ours is no exception. Some experimental data are clearly inconsistent with our predictions. For example, human cancer cells move faster on stiffer substrates, whereas our simulated cells are slower. For smooth muscle cells, it was reported that cell velocity has a biphasic dependence on substrate stiffness, i.e. cells move slowest on the softest and stiffest substrates (Peyton and Putnam, 2005). Clearly, real cells are much more complex than any model, and biochemical signaling acts to shape, regulate, and fine-tune the cell's mechanical sensing and response, aspects that are not yet considered in our model. A bridge between the mechanical and biochemical signals for cell migration is a promising avenue for future models.

For computational simplicity, our model represents the ECM as a uniform, isotropic, linearly elastic material (e.g., synthetic polyacrylamide gel), with fiber sizes that are sufficiently small relative to the size of the cell. So, our predictions are most accurate for cells moving on PA gels. In natural matrices, inhomogeneities result from traction-force-induced fiber realignment, density changes, and nonlinear strain-stiffening. As a first step toward more complex matrices, we have studied how cell spreading, elongation, and durotaxis are affected by inhomogeneity in substrate stiffness (uniform noise, Figures S4A–C) and explored the effect of small-scale spatial inhomogeneities on cell elongation (Figures S4D–L). In our ongoing work, we are extending the model to better represent the fibrous and strain-stiffening properties of natural ECMs (Hall et al., 2016; Han et al., 2018; van Helvert and Friedl, 2016) by incorporating discrete ECM models (Feng et al., 2015, 2018; Kim et al., 2017; Licup et al., 2015) with our cell-based models. Although the effect of traction forces propagates further into nonlinearly elastic, fibrous ECMs (Ma et al., 2013), the key results of our model are based on increased stability of FAs on strained matrices and so, will likely still apply.

Our model currently does not accurately predict that FAs are larger on stiff than on soft ECM substrates (Figure 2D) (Prager-Khoutorsky et al., 2011). Although there is a small increase in size in the range between 1 kPa and 10 kPa, for large stiffnesses the FAs do not increase in size. This might be due to the assumed fixed pool of free integrin bonds that reduces the growth rate of new FAs once many FAs already exist. Furthermore, our lattice-based model does not consider spatial, cooperative effects in integrin clustering: small clusters may merge into larger adhesions. Integrins diffuse along the cell membrane and they are activated upon interaction with regulatory proteins such as talin (Welf et al., 2012) and vinculin (Humphries et al., 2007). Similar mechanisms include phosphorylation of p130cas in response to stretching, which activates the small GTPase Rap1 (Sawada et al., 2006) and resulting integrin activation (Bos et al., 2003). Future versions of our model will consider these and further regulatory mechanisms of FA dynamics (Ali et al., 2011; Deshpande et al., 2008; Vernerey and Farsad, 2014; Welf et al., 2012) and thus refine its predictive value.

Another simplifying assumption concerns the distribution of the cellular traction forces (Lemmon and Romer, 2010). Although this model has been experimentally validated, it has some limitations, principally, that it is inconsistent with the Hamiltonian-based CPM forces. (The Hamiltonian acts as a potential energy, whose gradient defines a force field, at least along the cell edge; see Rens and Edelstein-Keshet (2019).) Alternative force descriptions have also been proposed in Albert and Schwarz (2014). These formulations also lead to different spatial distributions of forces inside the cell. In Rens and Edelstein-Keshet (2019), for example, it was shown that linear, polynomial, or exponential dependence of force on distance from the cell centroid were all consistent with experimental traction force data. In real cells, traction force distributions depend on the details of the stress fiber localizations and substrate topography (Soiné et al., 2015). In models that we have recently developed (Pomp et al., 2018; Schakenraad et al., 2020), cell geometry affects cytoskeletal orientation, which in turn determines the directionality of stress fibers. Integrating these new traction force models into the CPM will result in future improvements and greater accuracy.

### Limitations of Study

Our work is computational, and, although based on experimental literature, has yet to be (in)validated against dedicated future experimental studies. The model assumes a homogeneous linearly elastic ECM, among many simplifications. Hence, it falls short of explaining biological behavior that stems from the true fibrous, nonlinear elastic nature of the ECM. The model presently omits intracellular signaling cascades that sense and respond to mechanochemical stimuli and thus, in its current form, does not accurately account for every experimentally observed cell behavior nor is it calibrated to specific cell types. To apply our model to more specific cell systems, additional cellular and adhesion mechanisms and cell-extracellular matrix interactions should be considered. Finally, any modeling platform has limitations as well as benefits. Although the cellular Potts framework allows for highly resolved cell shape computations, it comes with drawbacks, and does not explicitly describe cell forces nor inherent cellular dynamics time scales.

### Resource Availability

#### Lead Contact

Further information and requests for resources and reagents should be directed to and will be fulfilled by the Lead Contact, Roeland M.H. Merks (merksrmhmath.leidenuniv.nl).

#### Materials Availability

There are no materials associated with this work.

#### Data and Code Availability

The model (C++ code) and parameter files discussed in the paper are available from GitHub at https://github.com/rmerks/FA-CPM-FEM. The code was written in the Cellular Potts—Finite Element framework described in van Oers et al. (2014).

## Methods

All methods can be found in the accompanying Transparent Methods supplemental file.

## Supplemental Information

Document S1. Transparent Methods, Figures S1–S6, and Tables S1 and S2
