# Supplementary material for: Cell Shape and Durotaxis Explained from Cell-Extracellular Matrix Forces and Focal Adhesion Dynamics
Source: iScience. 2020 Aug 22;23(9):101488. doi: 10.1016/j.isci.2020.101488 (PMC7482025; doi:10.1016/j.isci.2020.101488)
Supplement: Document S1. Transparent Methods, Figures S1–S6, and Tables S1 and S2 [file mmc1.pdf]

iScience, Volume *23*

Supplemental Information

Cell Shape and Durotaxis Explained from Cell-Extracellular Matrix Forces and Focal Adhesion Dynamics

Elisabeth G. Rens and Roeland M.H. Merks

Supplemental Figures

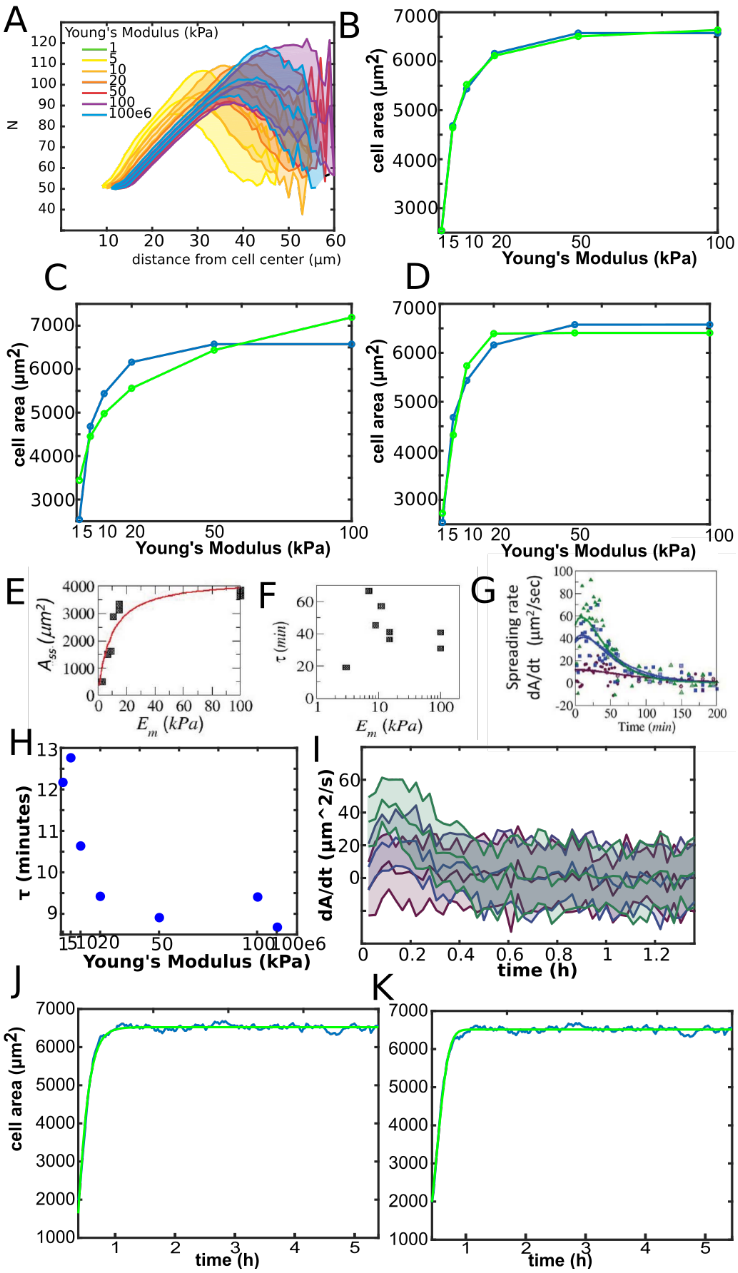

Figure S1: More detailed quantification of Model 1. Related to Figure 2. (A) The number of integrin bonds per cluster (N) as a function of distance from the cell center. All clusters at 2000 MCS from 25 simulations were pooled. Shaded regions show standard deviations. In B–D and J–K, phe-

nomenological functions are shown in green, and simulation data in blue. (B) The function $A(E) = \frac{a \cdot E^m}{K^m + E^m} + c$, proposed in Engler et al. (2004). (C) The function $A(E) = b \cdot E^n$, proposed in Engler et al. (2004). (D) The function $A(E) = a \cdot normcdf(E, \mu, \sigma)$, proposed in Balcioglu et al. (2015). (E,F,G): Experimental data from Nisenholz et al. (2014). (E) Steady state area as a function of substrate stiffness. (F) Spreading time-scale τ as a function of substrate stiffness, based on fitting $A(t) = A_0 \cdot (1 + R_0 \cdot (1 - \exp(-\frac{t}{\tau})))^2$. (G) Spreading rate as a function of time for cells on substrates of 7kPa (purple), 15kPa (blue) and 100kPa(green). (H-I) Simulation results. (H) Spreading time-scale τ as a function of substrate stiffness, based on the fit $A(t) = A_0 \cdot (1 + R_0 \cdot (1 - \exp(-\frac{t}{\tau})))^2$ proposed in Nisenholz et al. (2014). (I) Spreading rate as a function of time for cells on substrates of 1kPa (purple), 5kPa (blue) and 50kPa (green). (J) A function fit $A(t) = A_0 \cdot (1 + R_0 \cdot (1 - \exp(-\frac{t}{\tau})))^2$ proposed in Nisenholz et al. (2014) for a cell spreading on a substrate of 50kPa. (K) A function fit $A(t) = A_{50} \cdot (1 + erf(\frac{t - t_{50}}{\tau}))$ proposed in Reinhart-King et al. (2005) for a cell spreading on a substrate of 50kPa.

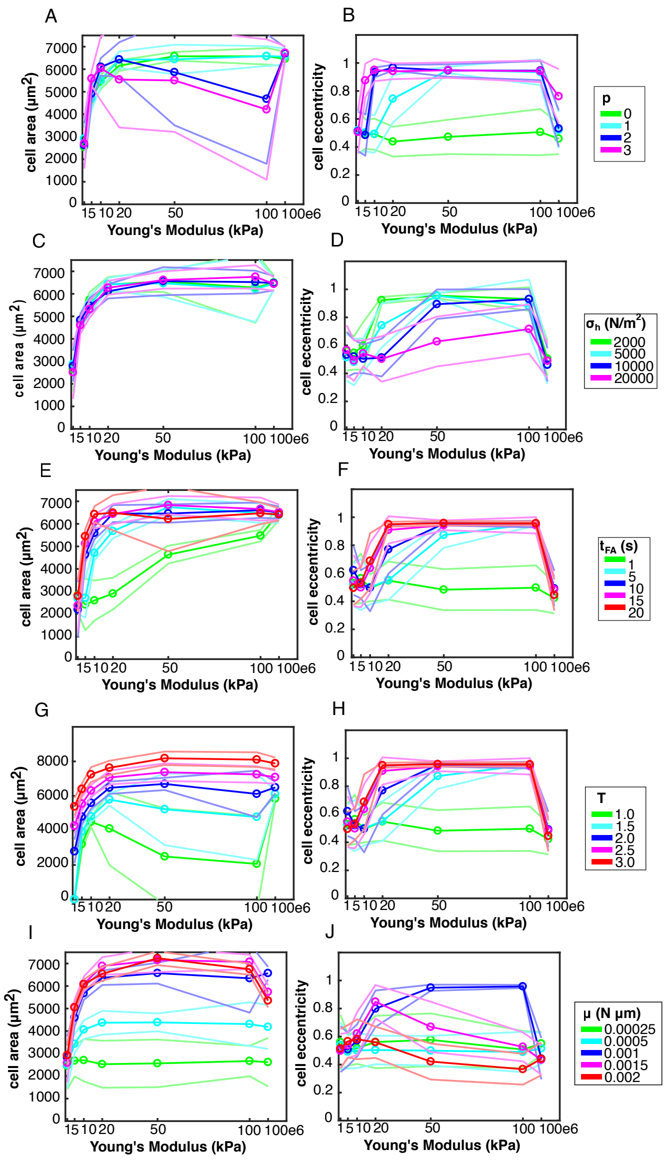

Figure S2: Model sensitivity of cell area and cell eccentricity to various parameters. Related to Figure 3. (A,B) Actin-integrin strength p. (C,D) saturation value for actin-integrin strength σ_h. (E,F) focal adhesion growth time t_{FA}. (G,H) cellular temperature T. (I,J) traction force magnitude μ. Shaded regions: standard deviations over 25 simulations.

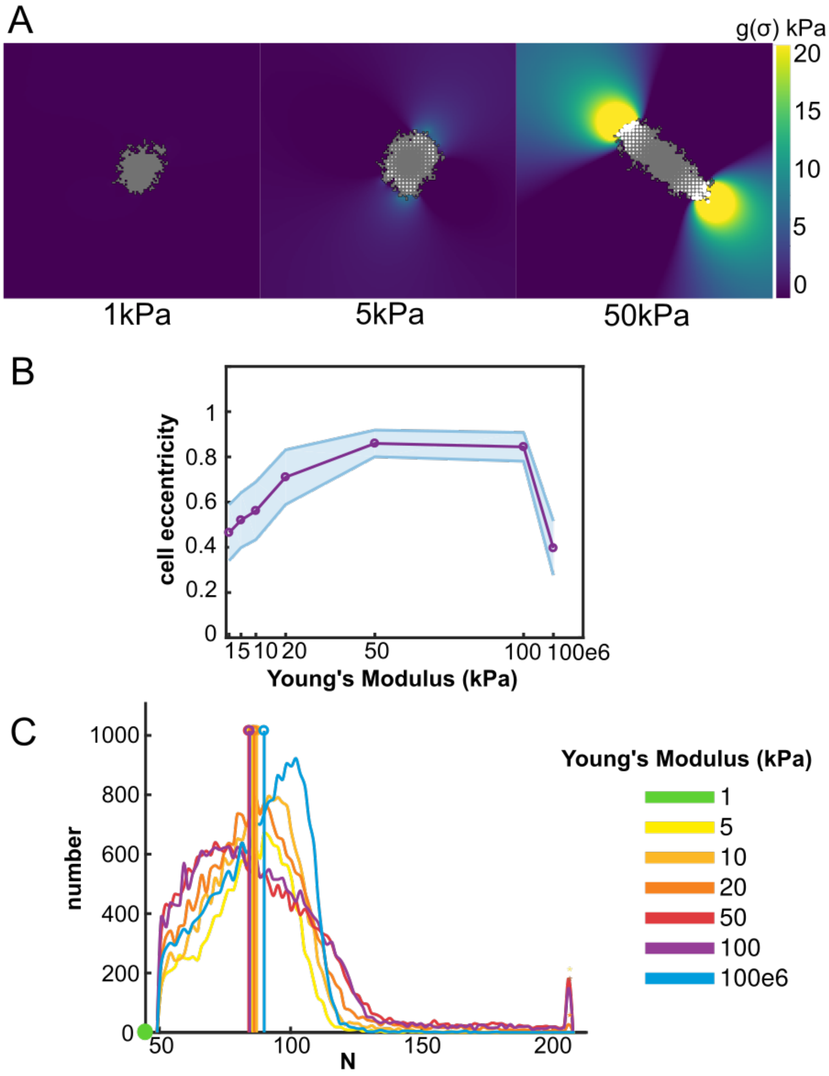

Figure S3: Cells elongate on substrates of intermediate stiffness. Related to Figure 3. Model 2.2 was used where matrix stress reinforces traction force $\vec{F}_s$ with $p = 5$. (A) Example configurations of cells at 2000 MCS on substrates of 1,50 and 50 kPa. Colors: hydrostatic stress; (B) Cell eccentricity as a function of substrate stiffness, shaded regions: standard deviations over 25 simulations; (C) distribution of N, the number of integrin bonds per cluster, all focal adhesion at 2000 MCS from 25 simulations were pooled. We indicate the median. Color coding (C): See legend next to (C).

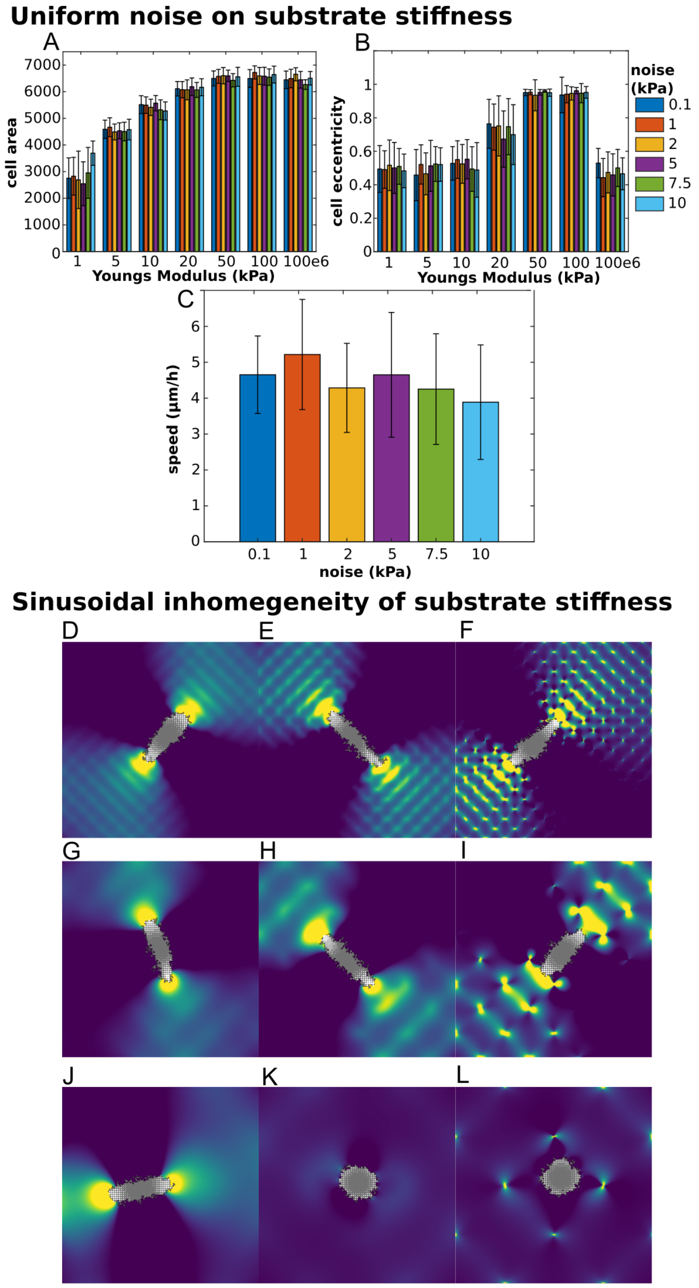

Figure S4: Cell behavior in presence of local variations in substrate stiffness. Related to Figure 2, 3 and 5. (A-C) Cell behavior on substrates with uniform noise in substrate stiffness, see Methods. (A) Cell area at 2000 MCS in Model 1. (B) Cell eccentricity at 2000 MCS in Model 2-version1. (C)

Durotaxis speed. Values: mean ± standard deviation over 25 simulations. (D-L) Cell elongation in Model 2-version 1 on a substrate of 50kPa with periodically varying substrate stiffness, see Methods. (D) $E_A = 10, T_E = 125\mu$m, (E) $E_A = 20, T_E = 125\mu$m, (F) $E_A = 50, T_E = 125\mu$m, (G) $E_A = 10, T_E = 312.5\mu$m, (H) $E_A = 20, T_E = 312.5\mu$m, (I) $E_A = 50, T_E = 312.5\mu$m, (J) $E_A = 10, T_E = 625\mu$m, (K) $E_A = 20, T_E = 625\mu$m, (L) $E_A = 50, T_E = 625\mu$m.

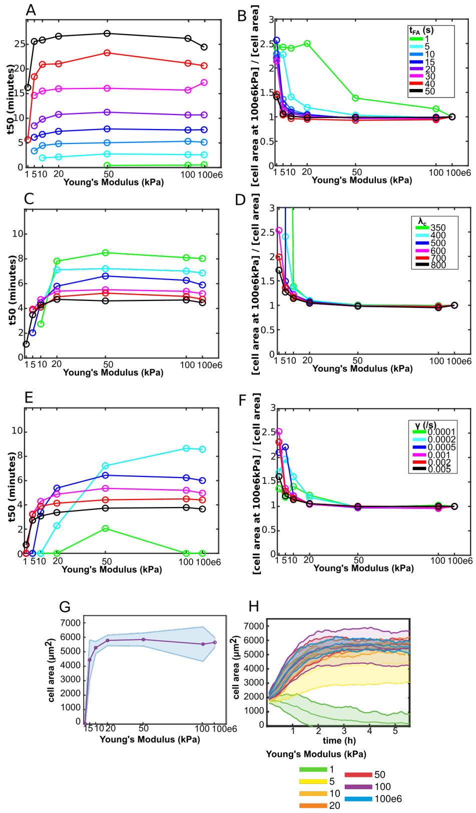

Figure S5: Sensitivity of cell area and spreading to various parameters. Related to Figure 2. (A,B) the time of assigned to one MCS (t_{FA}); (C,D) spreading strength (λ_C), and (E,F) the FA growth rate (γ). The time t_{50} is the time at which cells have spread to half their maximal spreading area. Similar to Reinhart-King et al. (2005) we calculate t_{50} by fitting the function $A_{50} \cdot (1 + erf\left(\frac{t - t_{50}}{\tau}\right))$ to the mean area of cells over time (see Fig2C). We also calculated the mean area of cells divided by the mean area of cells at the stiffest substrate (B,D and F), with default parameters showing a 2.5 fold increase of cell area on the stiffest substrate compared to the softest substrate. (G,H) Model 1 predictions with parameter changes $t_{\text{FA}} = 30s$, $\lambda_C = 600$, $\gamma = 0.005$. (G) Cell area as a function of substrate stiffness, shaded regions: standard deviations over 25 simulations; (H) Timeseries of cell area, shaded regions: standard deviations over 25 simulations.

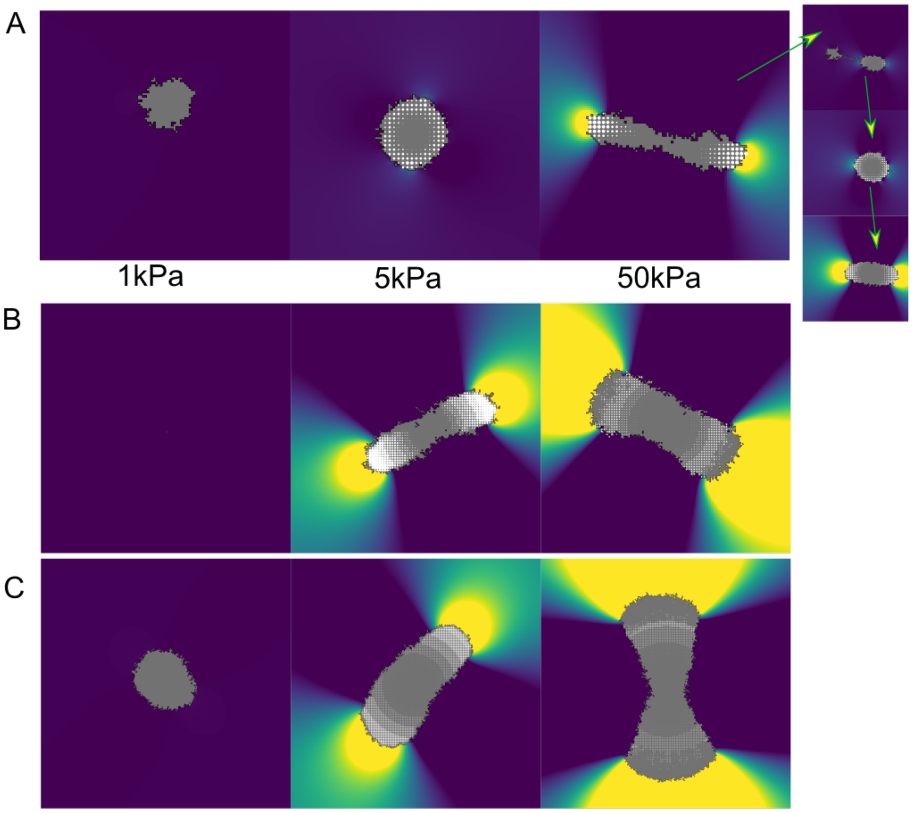

Figure S6: Rescaling of Model (version 2.1). Related to Figure 3. (A) Model results for lower temperature ($T = 0.5$). The spreading strength parameter λ_C was increased to 1000, otherwise cells would not spread. Notice that at this lower temperature, an elongated cell shape is unstable (see the three panels to the right): the cell repeatedly rounds up and elongates again. (B) Rescaling the grid by a factor of $s = 2$: $\Delta x = \Delta x/s, \lambda = \lambda/s^2, J = J/s, \lambda_C = \lambda_C/s^2, v_0 = v_0/s^2, \mu = \mu/s^2, N_0 = N_0/s^2, N_b = N_b/s^2, N_h = N_h/s^2, \gamma = \gamma/s^2$. (C) Rescaling the grid by a factor of $s = 4$. In (C), cells disappear at 1kPa (they become 1 pixel in size). In both (B) and (C), the elongated shape at 50kPa is unstable like in (A).

Supplemental Tables

Table S1: Parameter values. Related to Figure 2 to 5.

Symbol	Description	Value	Unit	Citation
Δx	lattice site width	2.5	μm	chosen
λ	area constraint/cell stiffness	0.0002	-	chosen
J	adhesive energy	7.5e-3	-	chosen
nbo	neighbourhood radius for adhesive energy	10	-	Magno et al. (2015)
λ_C	adhesion strength	600	-	chosen
A_h	half-max area	1000	lattice sites	chosen
λ_N	focal adhesion strength	4	-	chosen
N_h	half-max adhesion	200	-	chosen
p	actin-integrin strength	1	-	chosen
σ_h	half-max stress for actin-integrin binding	5000	N/m^2	chosen
T	cellular temperature	2	-	chosen
μ	traction magnitude	0.001	Nm/lattice site	estimated by traction stresses Califano and Reinhart-King (2010a)
v_0	free velocity of myosin molecules	100	nm/s	estimated by non-muscle myosin IIB Norstrom et al. (2010); Vogel et al. (2013)
E	Young's modulus	10000	N/m^2	varies
ν	Poisson's ratio	0.45	-	chosen
τ	substrate thickness	10	μm	Ambrosi (2006)
γ	growth rate	0.01	/s	estimated Novikova and Storm (2013)
d_0	unbinding rate	1	-	Novikova and Storm (2013)
N_0	size initial adhesion	50	-	nascent adhesion Changede and Sheetz (2016)
N_m	maximum free bonds	1e5	-	chosen Benedetto et al. (2006)
N_b	maximum size focal adhesion	390	-	chosen Changede and Sheetz (2016)
ϕ_s	slip tension	4.02	-	Novikova and Storm (2013)
ϕ_c	catch tension	7.76	-	Novikova and Storm (2013)
α	force scaling	0.01	$\frac{\#\text{integrins}}{N/m^2}$	estimated Novikova and Storm (2013)
t_{FA}	focal adhesion growth time	10	s	estimated by protrusion lifetimes Knorr et al. (2011)
Δt_{FA}	time steps	0.01	s	chosen

Table S2: Comparison of cell area vs stiffness curves to various experimental functions. Related to Figure 2.

Ref	Cell type	ECM type	Function	Experiment	Simulation
Engler et al. (2004)	SMC	collagen coated PA gel	$A(E) = b \cdot E^n$	$n = 0.29$	$n = 0.16$ (0.05391, 0.266)
				$b = 4000$ Pa	$b = 1140$ Pa (-95.13, 2376)
Engler et al. (2004)	SMC	collagen coated PA gel	$A(E) = \frac{a \cdot E^m}{K^m + E^m} + c$	$m = 0.87$	$m = 1.143$ (0.07275, 2.213)
				$K = 10000$ Pa	$K = 3591$ Pa (-398.2, 7581)
Balcioglu et al. (2015)	GDβ_3	PA gel functionalization with fibronectin	$A(E) = a \cdot normcdf(E, \mu, \sigma)$	$\mu = 2160$ Pa	$\mu = 2171$ Pa (-108.8, 4451)
				$\sigma = 2109$ Pa	$\sigma = 6242$ Pa (1812, 1.067e4)

Transparent Methods

We developed a multiscale model where cell movement depends on force induced focal adhesion dynamics. The model couples a cell-based model, substrate model and focal adhesion model in the following way. The Cellular Potts Model (CPM) describes cell movement. The shape of the cell is used to describe the stall forces that the cell exerts on the focal adhesions attached to a flexible substrate. These forces affect the growth of the focal adhesions. We assume that focal adhesions are clusters of integrins that behave as catch-slip bonds. Its dynamics are described using ordinary differential equations (ODEs). Finally, we assume that the cell-matrix link is strengthened by matrix stresses, which we calculate using a finite element model (FEM). The default parameter values are described in Table S1.

Cellular Potts Model

To simulate cell movement, we used the Cellular Potts Model (CPM) (Graner and Glazier, 1992). The CPM describes cells on a lattice $\Lambda \subset \mathbb{Z}^2$ as a set of connected lattice sites. Since the simulations in this article are limited to one cell, we describe the CPM here for a single cell. To each lattice site $\vec{x} \in \Lambda$ a spin $s(\vec{x}) \in \{0, 1\}$ is assigned. This spin value indicates if $\vec{x}$ is covered by the cell, $s(\vec{x}) = 1$, or not, $s(\vec{x}) = 0$. Thus the cell is given by the set,

$$C = \{\vec{x} \in \Lambda | s(\vec{x}) = 1\}. \tag{1}$$

The cell set C evolves by dynamic Monte Carlo simulation. During one Monte Carlo Step (MCS), the algorithm attempts copy a spin value $s(\vec{x})$ from a source site $\vec{x}$ into a neighboring target site $\vec{x}\prime$ from the usual Moore neighbourhood. Such copies mimic cellular protrusions and retractions. During an MCS, N copy attempts are made, with N the number of lattice sites in the lattice. Whether a copy is accepted or not depends on a balance of forces, which are represented in a Hamiltonian H.

A copy is accepted if H decreases, or with a Boltzmann probability otherwise, to allow for stochasticity of cell movements:

$$P(\Delta H) = \begin{cases} 1 & \text{if } \Delta H + Y < 0 \\ e^{-(\Delta H + Y)/T} & \text{if } \Delta H + Y \geq 0. \end{cases} \quad (2)$$

Here $\Delta H = H_{\text{after}} - H_{\text{before}}$ is the change in H due to copying, and the cellular temperature $T \geq 0$ determines the extent of random cell motility. Furthermore, Y denotes a yield energy, an energy a cell needs to overcome to make a movement. Finally, to prevent cells from splitting up into disconnected patches, we use a connectivity constraint that always rejects a copy if it would break apart a cell in two or more pieces.

Following Albert and Schwarz (2014), we use the following Hamiltonian:

$$H = \underbrace{\lambda A^2}_{\text{contraction}} + \underbrace{\sum_{\text{neighbours}(\vec{x},\vec{x}\prime)} J(s(\vec{x}), s(\vec{x}\prime))}_{\text{line tension}} - \underbrace{\lambda_C \frac{A}{A_h + A}}_{\text{cell-matrix adhesion}} . \quad (3)$$

The first term of H denotes cell contraction, where A is the area of the cell and λ is the corresponding Lagrange multiplier. In the second term, $J(s(\vec{x}), s(\vec{x}\prime))$ is the adhesive energy between two sites $\vec{x}$ and $\vec{x}\prime$ with spins $s(\vec{x})$ and $s(\vec{x}\prime)$. When taking a sufficient large neighborhood, this term describes a line tension, as it approximates the perimeter of a cell (Magno et al., 2015). We take a neighborhood radius of 10 for this calculation. The third term describes the formation of adhesive contacts of cells with the substrate, where the bond energies lower the total energy (Albert and Schwarz, 2014), causing the cells to spread. The parameter λ_{C} is the corresponding Lagrange multiplier. The energy gain of occupying more lattice sites saturates with the cell area, as the total number of binding sites is limited. The parameter A_h regulates this saturation.

To describe cell-matrix binding via focal adhesions, we implement the following yield energy in the CPM

$$Y = \lambda_{\text{N}} \frac{N(\vec{x}') - N_0}{N_h + N(\vec{x}')} \cdot \mathbf{1}_{s(\vec{x}')=1} \cdot \mathbf{1}_{s(\vec{x})=0}, \quad (4)$$

where $N(\vec{x}')$ is the size of the focal adhesion at the target site. This models that a retraction is energetically costly for a cell to make, because it needs to break the actin-integrin connection. We assume that the size of the actin-integrin link is proportional to the size of the focal adhesion, *i.e.* the number

of integrin bonds (Boettiger, 2007), and that the strength of a focal adhesion saturates (Gallant et al., 2005) with a parameter N_h. The substraction of N_0 represents that a focal adhesion only creates extra linkage if it is greater than a nascent adhesion. Note that the Y can not become negative, because we assume that focal adhesions smaller than N_0, a nascent adhesion, breaks down due to its short lifetime, see section 'Focal Adhesions'. So, only focal adhesions larger than N_0 create a yield energy. In section 'Substrate stresses', we further adapt this yield energy to describe a matrix stress induced focal adhesion reinforcement.

Cell traction forces

Following Schwarz et al. (2006), we assume that traction forces are generated by myosin molecular motors on the actin fibers, of which the velocity is given by

$$v(\vec{F}) = v_0 \left(1 - \vec{F}/\vec{F}_s\right), \tag{5}$$

where v_0 is a free velocity. The traction forces are applied to the ECM, which we assume is in plane stress. The constitutive equation is given by $h\vec{\nabla}\underline{\sigma} = \vec{F}$ where $\underline{\sigma}$ is the ECM stress tensor and h is the thickness of the ECM. We assume that the ECM is isotropic, uniform, linearly elastic and we assume infinitesimal strain theory. We solve this equation using a Finite Element Model (FEM) (see section 'Substrate stresses'). In the FEM, traction field $\vec{f}$ and ECM deformation $\vec{u}$ are related by:

$$\mathbf{K}\vec{u} = \vec{f}, \tag{6}$$

where $\mathbf{K}$ is the global stiffness matrix given by assemblying the local stiffness matrices $\mathbf{K}_e$ for each lattice site e

$$\mathbf{K}_e = h \int \mathbf{B}^T \frac{E}{1-\nu^2} \begin{pmatrix} 1 & \nu & 0 \\ \nu & 1 & 0 \\ 0 & 0 & \frac{1-\nu}{2} \end{pmatrix} \mathbf{B}, \tag{7}$$

where $\mathbf{B}$ is the conventional strain-displacement matrix for a four-noded quadrilateral element (Davies, 2011) and E is the Young's modulus and ν is the Poisson's ratio of the ECM. For more details on this part of the model, we refer to our previous work (van Oers et al., 2014; Rens and Merks, 2017).

Following Schwarz et al. (2006), the force build-up is given by the ODE:

$$\mathbf{K}\vec{v}(\vec{f}) = \frac{d\vec{f}}{dt}, \tag{8}$$

The matrix $\mathbf{K}$ describes force interactions between neighbouring nodes in the ECM. However, since solving this equation is expensive, we ignore the interactions between neighbouring sites, *i.e.*, we reduce $\mathbf{K}$ to a scalar for each site $\vec{x}$. This gives us,

$$\vec{F}(\vec{x}, t) = \vec{F}_s(\vec{x}) + (\vec{F}_0(\vec{x}) - \vec{F}_s(\vec{x})) \exp(-t/t_k), \tag{9}$$

where $\vec{F}_0$ is the force already exerted by the actin and $t_k = \frac{|\vec{F}_s|}{v_0 K}$. Here, K is given by the diagonal entry of $\mathbf{K}$ at site $\vec{x}$, i.e. the stiffness of this node, neglecting changes in local stiffness due to connections to neighbouring nodes described in the off-diagonal entries of $\mathbf{K}$. Since the cell configuration and therefore the traction forces change each MCS, the tension on the focal adhesions does not build up from zero, but from the tension that was built up during the previous MCS: $\vec{F}_0$ at the current MCS is given by $\vec{F}(t_{\mathrm{FA}})$ of the previous MCS.

To calculate the stall force of the actin fibers, $\vec{F}_s$, we employ the empirical first-moment-of-area (FMA) model (Lemmon and Romer, 2010). This model infers the stall forces from the shape of the cell of the CPM, based on the assumption that a network of actin fibers in the cell acts as a single, cohesive unit,

$$\vec{F}_s(\vec{x}) = \frac{\mu}{A} \sum_{\{\vec{y} \in C | [\vec{y}\vec{x}] \subset C\}} \vec{x} - \vec{y}. \tag{10}$$

So, the force at site $\vec{x}$ is calculated as the sum of forces between $\vec{x}$ and all other sites $\vec{y}$ within cell C that are connected to $\vec{x}$ (this sum excludes line segments $[\vec{y}\vec{x}]$ running outside the cell that occur if the shape of cell C is non-convex). The force is assumed to be proportional to the distance between the sites. We divide over the cell area A such that force increases roughly linear with cell area, as experimentally observed (Califano and Reinhart-King, 2010b).

Focal adhesions

At each lattice site occupied by the cell, $\vec{x} \in C$, a focal adhesion is modeled as a cluster of bound integrin bonds N. Each individual integrin bond behaves as a catch-slip bond, whose lifetime is maximal under a positive force (Novikova and Storm, 2013). Accordingly, the growth of a cluster of such bonds is described by the ODE-model derived by Novikova and Storm (2013),

$$\frac{dN(\vec{x},t)}{dt} = \gamma N_a(t) \left(1 - \frac{N(\vec{x},t)}{N_b}\right) - d(\phi(\vec{x},t), N(\vec{x},t) \cdot N(\vec{x},t) \tag{11}$$

with γ is the binding rate of integrins to the ECM, N_a the number of free bonds, and N_b the maximum number of bound bonds a lattice site can contain. Following the previous work (Sun et al., 2009, 2011; Novikova and Storm, 2013), we assume that the closing of integrin bonds occurs at a constant rate once the first adhesion bonds bring the cell membrane in sufficiently close proximity to the ECM; we assume that this is the case when the cell covers the lattice site. The logistic growth term, $\gamma N_a(t) \left(1 - \frac{N(\vec{x},t)}{N_b}\right)$, is a slight modification of the previous work (Novikova and Storm, 2013) and avoids packing more integrins in a lattice site than the size of the lattice site (Δx^2) can accommodate.

The degradation of the focal adhesions $d(\phi)$ depends on the tension ϕ on the focal adhesion N. This degradation rate is given by

$$d(\phi(\vec{x},t), N(\vec{x},t)) = d_0 \left[\exp\left(\frac{\alpha\phi(\vec{x},t)}{N(\vec{x},t)} - \phi_s\right) + \exp\left(-\frac{\alpha\phi(\vec{x},t)}{N(\vec{x},t)} + \phi_c\right)\right] \tag{12}$$

where ϕ_s and ϕ_c are nondimensional parameters that describe the slip and catch bond regime, respectively. Parameter d_0 is the baseline unbinding rate of the integrins Novikova and Storm (2013). Here, $\phi(\vec{x},t) = \frac{|\vec{F}(\vec{x},t)|}{\Delta x^2}$ is the stress applied to the lattice site of the focal adhesion. To conform the units of force $\phi(\vec{x},t)$ to the dimensionless parameters ϕ_s and ϕ_c, we scale it with α (units $\frac{\#\text{integrins}}{N/m^2}$) in Equation 12. We assume that the number of free bonds N_a is limited by the number of available integrin receptors in the entire cell, N_m. These N_m receptors can be recruited to each focal adhesion site and enable binding of a bond. Thus, $N_a(t) = N_m - \sum_{\vec{x}\in C} N(\vec{x},t)$. We let the focal adhesions grow after each MCS for t_{FA} seconds with time increments of Δt_{FA}. If there is no pre-existing focal adhesion at site $\vec{x} \in C$, we set $N(\vec{x}) = N_0$, so that at this site, a new initial adhesion is formed. This assumption represents the generation of focal complexes or nascent adhesions, precursors of focal adhesions that contain a small amount of integrins and have a very short lifetime(Changede and Sheetz, 2016). Also, after the focal adhesions were allowed to grow, *i.e.* after $t = t_{\text{FA}}$ seconds, we set all $N(\vec{x}) < N_0$ back to $N(\vec{x}) = N_0$, again modeling the quick (re)generation of nascent adhesions.

When a site $\vec{x}$ is removed from the cell C after a retraction, such that $s(\vec{x}) = 0$, we set $N(\vec{x}) = 0$ reflecting the destruction of the focal adhesion. We assume that if a cell extends, i.e. a site $\vec{x}$ is added to the cell C, a nascent adhesion is formed: we set $N(\vec{x}) = N_0$.

Substrate stresses

The forces that were build up during a MCS, $\vec{F}(t_{\text{FA}})$ are applied as planar forces to a finite element model (FEM). The FEM calculates the stress tensor $\underline{\sigma}(\vec{x})$ on each lattice site. We assume that the integrin-cytoskeletal adhesion strengthens as a result of stress. We define

$$g(\underline{\sigma}) = \begin{cases} \frac{1}{2}(\sigma_{xx} + \sigma_{yy}) & \text{if } \frac{1}{2}(\sigma_{xx} + \sigma_{yy}) \geq 0 \\ 0 & \text{if } \frac{1}{2}(\sigma_{xx} + \sigma_{yy}) < 0 \end{cases} \tag{13}$$

the positive hydrostatic stress of the stress tensor that describes how much stress the focal adhesion experiences.

Model 2.1

We extend the yield energy as follows:

$$Y = \lambda_{\text{N}} \frac{N(\vec{x}') - N_0}{N_h + N(\vec{x}')} \cdot \left(1 + p\frac{g(\underline{\sigma}(\vec{x}'))}{\sigma_h + g(\underline{\sigma}(\vec{x}'))}\right) \cdot \mathbf{1}_{\vec{x}' \in C} \cdot \mathbf{1}_{\vec{x} \notin C} \tag{14}$$

We thus assume that stress strengthens the focal adhesions, with parameter p and that this strengthening saturates with parameter σ_h.

Model 2.2

In model 2.1, we proposed that matrix stress induces focal adhesion strengthening but noted that matrix stress might also reinforce cell contractility. Supplementary Figure3 shows the results of having $\vec{F}_s = \vec{F}_s \cdot \left(1 + p\frac{g(\underline{\sigma}(\vec{x}'))}{\sigma_h + g(\underline{\sigma}(\vec{x}'))}\right)$ instead of focal adhesion strengthening as described in the main text. Since matrix stresses are defined on the lattice sites while forces are defined on the nodes of the lattice, we needed to assume some interpolation. We choose to take

$$\vec{F}_s = \vec{F}_s \cdot \frac{1}{4} \sum_{\text{surrounding4nodes}} \left(1 + p\frac{g(\underline{\sigma}(\vec{x}'))}{\sigma_h + g(\underline{\sigma}(\vec{x}'))}\right). \tag{15}$$

Stiffness gradient

To study durotaxis, we model a stiffness gradient in the x-direction on a lattice of 1250 μm by 500 μm. The Young's modulus of the substrate $E(Pa)$ is given by $E(x) = \max\{1, 6000 + (x - 250) \cdot \text{slope}\}$, with x in μm, such that the Young's modulus at the center of the cell at time $t = 0$ is 6000 Pa and is nonzero. The default value for the slope is 20 Pa/μm.

Noise in substrate stiffness

To simulate inhomogeneity in substrate stiffness, we add random noise between $-E_{\text{noise}}$ and $+E_{\text{noise}}$ to the Young's modulus of the substrate E at each lattice site. If this sets the substrate stiffness to a negative value, we cut it off at zero. We tested the effect of noise on cell spreading, cell elongation and durotaxis for values of E_{noise} of 100 Pa, 1 kPa, 2 kPa, 5 kPa, 7.5 kPa and 10 kPa. The results are in Supplementary Figure4.

Local variations in substrate stiffness

To simulate longer range inhomogeneities in substrate stiffness, we modify the substrate stiffness as follows:

$$E = E + E_A \cdot \cos(\frac{2\pi x}{T_E}) \cdot \cos(\frac{2\pi y}{T_E}) \tag{16}$$

where E_A is the amplitude of the sinusoidal and T_E its period. The results are in Supplementary Figure4.

Grid resolution

The spatial resolution of the cell and the number of integrins per CPM pixel can be changed by scaling Δx. In Figure S6B and C, we show cell elongation for rescaling by a factor of 2 and 4 respectively. The caption indicates the rescaling of the parameters. The results are qualitatively similar, although cells start to elongate at softer substrates and at 50kPa, the elongated shape is unstable (as in Figure S6A). On 1kPa, the cell fails to spread at all for a grid scaling factor of 2 (Figure S6B). Finally, the pool of integrins is also more quickly depleted, resulting in less focal adhesions near the cell edge. This causes instability of the elongated shape. When a cell is very long, its inward contractile force cannot be counteracted by the focal adhesion far from the cell edge.

References

Albert, P.J., Schwarz, U.S., 2014. Dynamics of cell shape and forces on micropatterned substrates predicted by a cellular potts model. Biophys. J. 106, 2340–2352.

Ambrosi, D., 2006. Cellular traction as an inverse problem. SIAM J. Appl. Math. 66, 2049–2060.

Balcioglu, H.E., van Hoorn, H., Donato, D.M., Schmidt, T., Danen, E.H.J., 2015. The integrin expression profile modulates orientation and dynamics of force transmission at cell–matrix adhesions. J. Cell Sci. 128, 1316–1326.

Benedetto, S., Pulito, R., Crich, S.G., Tarone, G., Aime, S., Silengo, L., Hamm, J., 2006. Quantification of the expression level of integrin receptor αvβ3 in cell lines and mr imaging with antibody-coated iron oxide particles. Magn Reson Med 56, 711–716.

Boettiger, D., 2007. Quantitative measurements of integrin-mediated adhesion to extracellular matrix. Methods Enzymol. 426, 1–25.

Califano, J.P., Reinhart-King, C.A., 2010a. Substrate stiffness and cell area predict cellular traction stresses in single cells and cells in contact. Cell. Mol. Bioeng. 3, 68–75.

Califano, J.P., Reinhart-King, C.A., 2010b. Substrate stiffness and cell area predict cellular traction stresses in single cells and cells in contact. Cell. Mol. Bioeng. 3, 68–75.

Changede, R., Sheetz, M., 2016. Prospects & overviews integrin and cadherin clusters: A robust way to organize adhesions for cell mechanics. Bioessays 93, 1–12.

Davies, A.J., 2011. The Finite Element Method: An Introduction With Partial Differential Equations. Oxford University Press, Oxford.

Engler, A., Bacakova, L., Newman, C., Hategan, A., Griffin, M., Discher, D., 2004. Substrate compliance versus ligand density in cell on gel responses. Biophys. J. 86, 617–628.

Gallant, N.D., Michael, K.E., Garcı, J., 2005. Cell adhesion strengthening: Contributions of adhesive area, integrin binding, and focal adhesion assembly. Mol. Biol. Cell. 16, 4329–4340.

Graner, F., Glazier, J.A., 1992. Simulation of biological cell sorting using a two-dimensional extended potts model. Phys. Rev. Lett. 69, 2013–2016.

Knorr, M., Koch, D., Fuhs, T., Behn, U., Kas, J.A., 2011. Stochastic actin dynamics in lamellipodia reveal parameter space for cell type classification. Soft Matter 7, 3192–3203.

Lemmon, C.A., Romer, L.H., 2010. A predictive model of cell traction forces based on cell geometry. Biophys. J. 99, L78–L80.

Magno, R., Grieneisen, V.A., Marée, A.F.M., 2015. The biophysical nature of cells: Potential cell behaviours revealed by analytical and computational studies of cell surface mechanics. BMC Biophys. 8.

Nisenholz, N., Rajendran, K., Dang, Q., Chen, H., Kemkemer, R., Krishnan, R., Zemel, A., 2014. Active mechanics and dynamics of cell spreading on elastic substrates. Soft matter 10, 7234–7246.

Norstrom, M., Smithback, P., Rock, R., 2010. Unconventional processive mechanics of non-muscle myosin ii-b. J. Biol 285, 26326–26334.

Novikova, E.A., Storm, C., 2013. Contractile fibers and catch-bond clusters: A biological force sensor? Biophys. J. 105, 1336–1345.

van Oers, R.F.M., Rens, E.G., LaValley, D.J., Reinhart-King, C.A., Merks, R.M.H., 2014. Mechanical cell-matrix feedback explains pairwise and collective endothelial cell behavior in vitro. PLoS Comput. Biol. 10, e1003774.

Reinhart-King, C.A., Dembo, M., Hammer, D.A., 2005. The dynamics and mechanics of endothelial cell spreading. Biophys. J. 89, 676–689.

Rens, E.G., Merks, R.M.H., 2017. Cell contractility facilitates alignment of cells and tissues to static uniaxial stretch. Biophys. J. 112, 755–766.

Schwarz, U.S., Erdmann, T., Bischofs, I.B., 2006. Focal adhesions as mechanosensors: The two-spring model. Biosystems 83, 225–232.

Sun, L., Cheng, Q.H., Gao, H.J., Zhang, Y.W., 2009. Computational modeling for cell spreading on a substrate mediated by specific interactions, long-range recruiting interactions, and diffusion of binders. Phys Rev E 79, 3583.

Sun, L., Cheng, Q.H., Gao, H.J., Zhang, Y.W., 2011. Effect of loading conditions on the dissociation behaviour of catch bond clusters. J. R. Soc. Interface 9, 928–937.

Vogel, S.K., Petrasek, Z., Heinemann, F., Schwille, P., 2013. Myosin motors fragment and compact membrane-bound actin filaments. Elife 2, e00116.
